# Analysis of The Reproducibility of Subgingival Vertical Margins Using Intraoral Optical Scanning (IOS): A Randomized Controlled Pilot Trial

**DOI:** 10.3390/jcm10050941

**Published:** 2021-03-01

**Authors:** Edoardo Ferrari Cagidiaco, Fernando Zarone, Nicola Discepoli, Tim Joda, Marco Ferrari

**Affiliations:** 1 Department of Periodontics, Complutense University, 28001 Madrid, Spain; edoardo.ferrari.cagidiaco@gmail.com; 2 Department of Prosthodontic and Dental Materials, University of Siena, 53100 Siena, Italy; 3 Department of Neurosciences, Reproductive and Odontostomatological Sciences, University of Naples Federico II, 80131 Napoli, Italy; fernandozarone@mac.com; 4 Department of Periodontics, University of Siena, 53100 Siena, Italy; ndiscepoli@me.com; 5 Department of Reconstructive Dentistry, University Center for Dental Medicine Basel, CH-4058 Basel, Switzerland; tim.joda@unibas.ch; 6 Department of Prosthodontics and Dental Materials, University of Siena, 53100 Siena, Italy

**Keywords:** knife-edge preparation, IOS, superimposition, digital impression, subgingival margins

## Abstract

Background: The aim of this randomized controlled trial was to evaluate the capability of an IOS (Intra Oral Scanner) device, used in standardized conditions, to detect margins of abutments prepared with knife-edge finishing line located at three different levels in relation to the gingival sulcus. Methods: sixty abutment teeth for treatment with full crowns were selected and randomly divided in three groups accordingly to the depth of the finishing line: Group A: supragingival margin; Group B: 0.5–1.0 mm into the sulcus; Group C: 1.5–2.0 mm into the sulcus. Temporary crowns were placed for two weeks and then digital impressions (Aadva IOS 100, GC, Japan) were made of each abutment. As controls, analog impressions were taken, poured, and scanned using a laboratory scanner (Aadva lab scanner, GC, Japan). Two standard tessellation language (STL) files were generated for each abutment, subsequently processed, and superimposed by Exocad software (Exocad GmbH, Darmstadt, Germany), applying the “best-fit“ algorithm in order to align the scan of the conventional with the digital impressions. The distances between each preparation margin and the adjacent gingival tissue were measured. Four measures were taken, two interproximally and buccally, for a total of six measures of each abutment considering three modes of impressions. The data were statistically evaluated using two-way analysis of variance (ANOVA) for each site and the Bonferroni test. Results: there was no difference between the two kinds of impression in Group A in both sites, in Group B a difference of 0.483 mm and 0.682 mm at interproximal and buccal sites, respectively, and in Group C 0.750 mm and 0.964 mm at interproximal and buccal sites, respectively. The analysis performed on a site level (mesial/distal/vestibular) for the depth of both vertical preparations revealed significant differences (*p* < 0.0001). After a post hoc analysis (Bonferroni), vestibular sites of the shallow vertical preparations resulted in significantly lower values compared to the other sites prepared deeply. Conclusions: the results showed that the location of the margin is an important factor in making a precise and complete impression when IOS (Intra Oral Scanner) is used. Moreover, deep preparation into the sulcus is not recommended for IOS (Intra Oral Scanner) impressions.

## 1. Introduction

Key factors for long-term clinical success in fixed prosthodontics are respect of function, biocompatibility, marginal and internal fit, fracture resistance, and appealing esthetics. In particular, a marginal gap, at the level of the restorative finish line, has a highly detrimental effect on the quality of the restoration, inducing micro-leakage, cement dissolution by oral fluids, and biofilm accumulation, with consequences such as caries or endodontic and periodontal problems [1,2,3,4]. Up to now, the precision of marginal fit has been reported up to 200 microns and beyond [5,6,7,8], although a precise, scientifically validated evaluation of the maximum acceptable marginal gap has never been provided; the threshold of 120 microns, defined by McLean, has been considered as a reference in dental literature since 1971 [9]. It is generally accepted that all subsequent clinical and laboratory work steps influence the overall success of a fixed restoration, from tooth preparation to cementation [10]. Here, the final impression is one of the most important steps to achieving the final marginal adaptation of the restoration, independent on the material and technique selected. In conventional impression procedures, the final result is strongly affected by dimensional distortions of impression materials and gypsum [11,12], to the extent that half of misfits have been considered to be ascribed to the impression procedure and to the production of the gypsum cast, the other half being mainly related to the production techniques of the prosthesis [13,14]. The introduction of the digital impression by using intraoral scanning (IOS) has changed the restorative scenario in prosthodontics by the acquisition of anatomic information without the use of physical impression materials, transforming shapes into digital files [15,16,17,18].

One of the most critical steps during impression taking, both conventional and digital, is detecting the finish line, in particular for subgingival tooth preparation. In this context, adequate soft tissue management without inflammation is mandatory for a successful impression, supported by gingival displacement to expose the finish. In the conventional impression procedure, this is usually obtained using gingival retraction cords or materials which temporarily modify the marginal soft tissue, with the purpose of detecting the necessary sub-gingival anatomic information and of widening the gingival sulcus without tearing the subtle light material margin, due to its low consistency [19]. Following the digital impression technique, it is not different to the conventional approach. In both cases, the detection of the finish line relies on a clean, healthy gingival sulcus, proper soft tissue displacement, and clear visibility of the prepared tooth anatomy.

The aim of this randomized controlled clinical trial was to test the capability of an IOS device (Aadva IOS 100, GC, Japan) used in standardized conditions, to detect margins of abutments prepared with knife-edge finishing line located at three different levels in relation to the gingival sulcus.

The null hypothesis was that there was no difference in the capability of the IOS based on the vertical position of the prepared finish line.

## 2. Experimental Section

In this study, 60 patients (28 female and 32 male) with a mean age of 45 (±20.5) years (range 18–69) in need of a tooth-borne single crown in posterior sites were recruited. The present prospective clinical trial was approved by the Ethical Committee of the University of Siena (n.18895). For each included individual, a signed written consent was obtained after clear information about the study. Guidelines of the CONSORT statement were followed.

Inclusion Criteria: age ≥ 18 years; single full crown in posterior sites (maxilla or mandible); periodontally healthy or successfully treated; general good health.

Exclusion criteria: presence of any active infection; severe periodontal inflammation; presence of chronic systemic disease; smoking more than 15 cigarettes per day; bruxism habits.

### 2.1. Randomization/Allocation Concealment/Masking of Examiners

Included patients were recruited between May and November of 2018 in the Department of Fixed Prosthodontics at the University of Siena and randomly divided into three groups of twenty each (3 × *n* = 20) according to the location depth of the finishing line made on the prepared abutments in relation to the sulcus:

Group A: supragingival margin.

Group B: margin 0.5–1.0 mm into the sulcus.

Group C: margin 1.5–2.00 mm into the sulcus.

**Figure 1:**
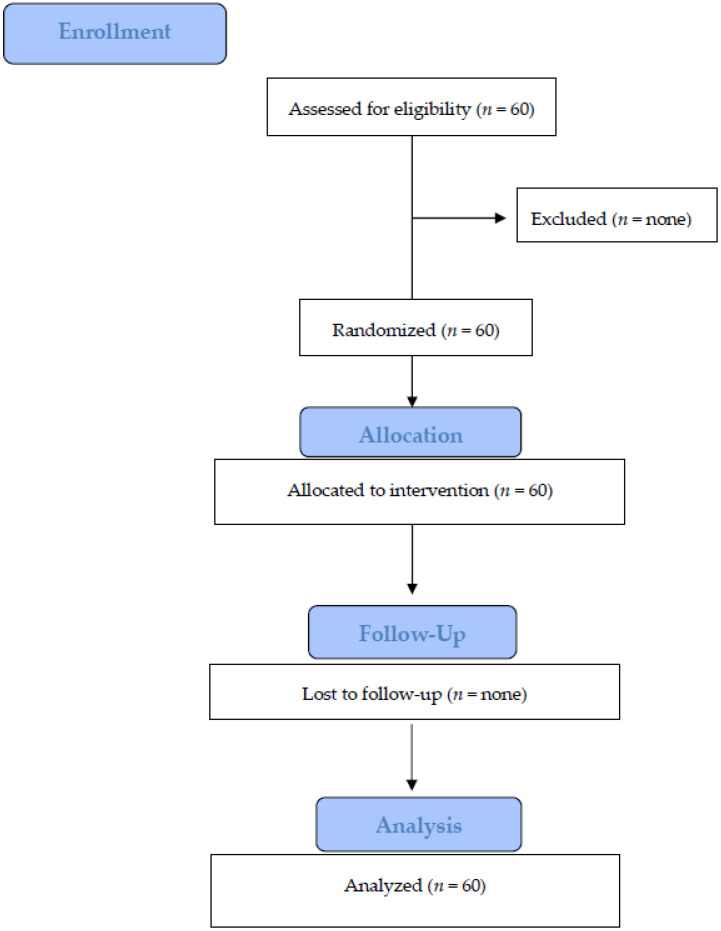
Flow diagram.

Treatment assignment was noted in a detailed registration and treatment assignment form. Allocation concealment was performed by opaque, sealed, and sequentially numbered envelopes. The statistician generated the allocation sequence by means of a computer-generated random list and instructed a different subject to assign a sealed envelope containing the type of IOS. The opaque envelope was opened before IOS selection and communicated to the operator (EFC—Edoardo Ferrari Cagidiaco). Blinding of the examiner was maintained throughout all experimental procedures (Figure 1).

### 2.2. Clinical Setting

**Figure 2:**
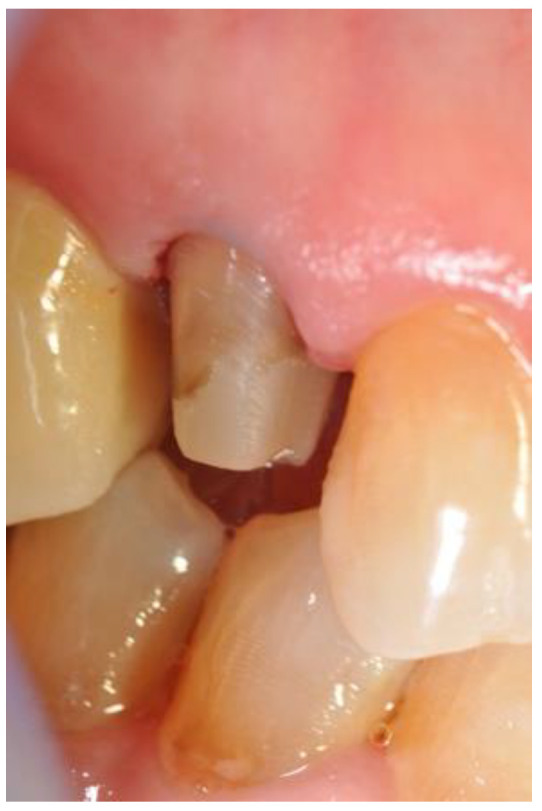
The abutment after preparation.

Abutment tooth preparations of Group A were performed following the generally accepted recommendations for CAD/CAM (Computer-Aided Design/Computer-Aided Manufacturing)-restorations with supragingivally located margins in order to remain visible [20]. In Group B, the margins were placed 0.5–1.0 mm into the sulcus and in Group C, the margins were placed around 1.5–2.0 mm in depth. Clinical pictures were taken of each quadrant and the corresponding preparations (Figure 2).

**Figure 3:**
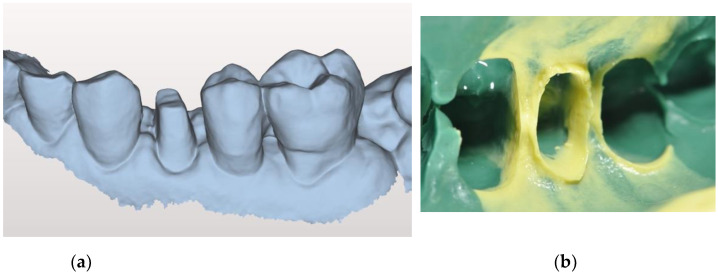
Digital impression (**a**)Analogic impression. The deep preparation is evident (**b**).

All abutments received a temporary crown for 2 weeks [21,22] and then the final IOS impressions were made. The impression site was prepared according to the double retraction cord technique: the first, thinner cord (Ultrapack #00; Ultradent, South Jordan, UT, USA) was gently placed into the gingival sulcus, followed by the insertion of a second, wider-diameter cord (Ultrapack #1; Ultradent, South Jordan, UT, USA) at a more coronal level, visible around the preparation margins. IOS was initially performed according to the manufacturer’s guidelines (Aadva IOS 100, GC Co., Tokyo, Japan): firstly, the upper arch was scanned, followed by the lower arch, and then the bite registration was performed. A total of twenty scans of each group (A, B, and C) were collected and saved in the standard tessellation language (STL) format (Figure 3a).

Any scanning shot considered incorrect or showing evident defects was discarded.

As the control, a conventional impression was made using polyvinyl siloxane (Ex’lance, GC) (Figure 3b).

The viscoelastic properties of the material facilitate the detection of the area below the gingival margins. Impressions were cleansed, disinfected, poured in Type IV Dental Die Stone (FujiRock, GC, Tokyo, Japan), and finally scanned by a laboratory scanner (Aadva lab scanner, GC, Tokyo, Japan), generating STL files of the control protocol.

### 2.3. Software Measurements

**Figure 4:**
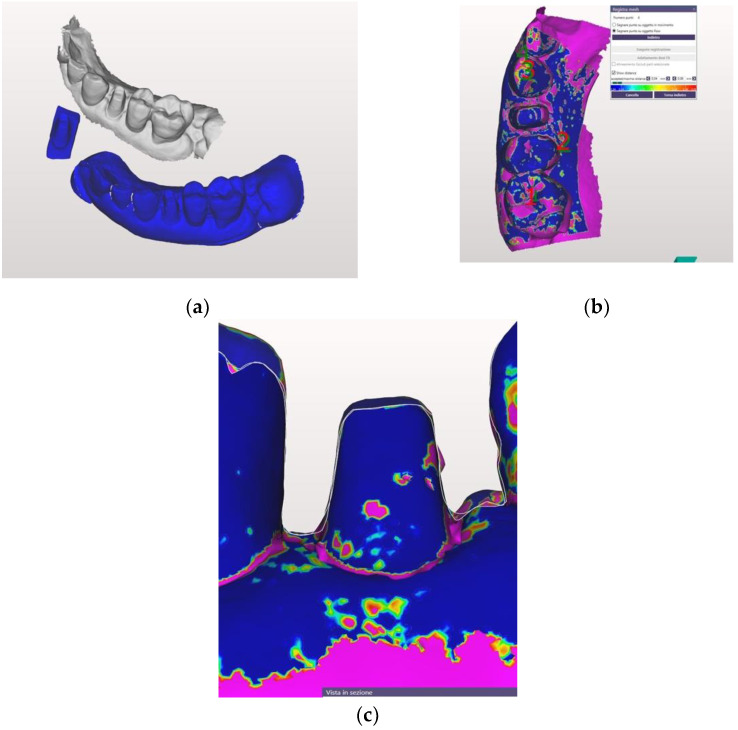
The two digital casts before being superimposed (**a**). The two digital casts after being superimposed (**b**). The abutment after being sovraimposed (**c**)**.**

Each STL file generated by both the IOS and the lab scanner was processed by the same dental master technician, using the Exocad software (Exocad GmbH, Darmstadt, Germany), applying the “best-fit“ algorithm in order to align the scan of the conventional with the digital impression (Figure 4a).

The superimposition of the STL files allowed measurement of the distance between each preparation margin and the adjacent gingival tissue, after making a section of each abutment in either the mesial-distal or buccal-lingual direction (Figure 4b,c).

The straight distance between the most coronal part of the gingival margin and the apical finish line of the preparation were used as distances to be recorded, and both vertical distances (made by conventional and digital impressions) were measured and recorded. The most coronal part of the gingival tissue was always the same, and the most apical part into the sulcus varied accordingly for each impression. Four measures were taken, two interproximally (mesial and distal) and buccally (buccal), for a total of six measures of each abutment considering three modes of impressions.

### 2.4. Statistical Analysis

All the data were collected and processed statistically. Descriptive statistics (means, standard deviations, 95% confidence intervals) were performed on the studied parameters using Stata 15-IC (IBM, NY, USA). The Wilcoxon rank sum test was used to analyze the media each measure.

Two-way analysis of variance (ANOVA) for each site and the Bonferroni test were conducted to assess the overall statistical significance of the differences among the groups (*p* > 0.05).

## 3. Results

Table 1 shows the results for the mean distance of the prepared root that cannot be detected with the digital impression compared to the conventional one.

**Figure 5:**
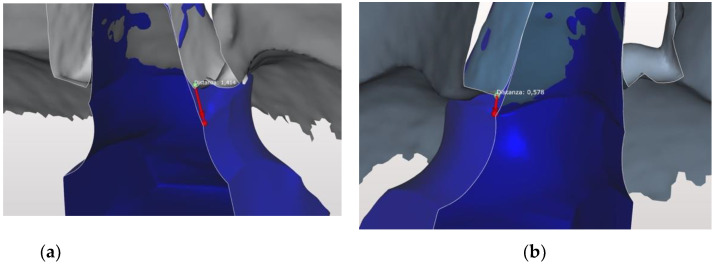
Differences in preparation reading of the two impressions in the mesial and distal areas (**a**,**b**).

**Figure 6:**
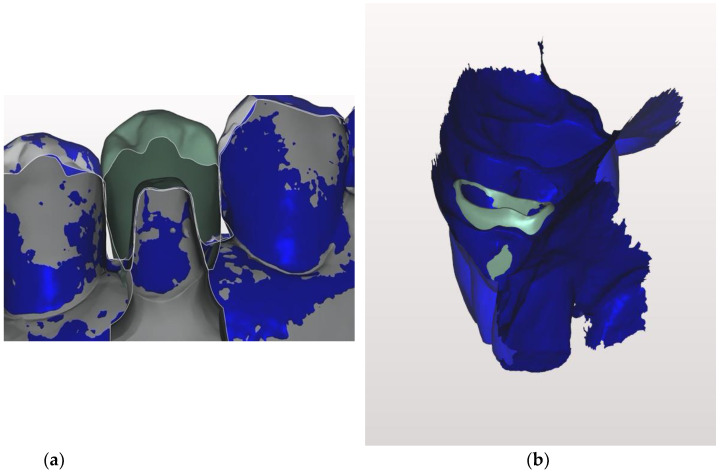
After waxing up the two crowns it is evident the difference in depth (**a**,**b**).

There was no difference between the two kinds of impression in Group A in both sites, in Group B a difference of 0.483 mm and 0.682 mm at the interproximal and buccal site respectively, and in Group C 0.750 mm and 0.964 mm at the interproximal and buccal site respectively (Figure 5a,b and Figure 6a,b).

The difference between the depth of the sulci, analyzed according to the two vertical preparations (Group B /<1 mm vs. Group C /1.5–2.0 mm), was statistically significant, with a difference of 0.28 mm (SE—Standard Error: 0.5; IC—Interval of Confidence: 95% −0.4–0.2) (*p* < 0.00).

The analysis performed on a site level (mesial/distal/vestibular) on the depth of both vertical preparations revealed significant differences (F = 12.15; *p* < 0.0001) (Table 2 and Table 3). After a post hoc analysis (Bonferroni) the vestibular site of the Group B vertical preparation was always statistically inferior to the other sites prepared deeply (Group C) (Table 4 and Table 5). 

The number of intraoral scans rejected from the study due to evident errors was 2 for Group A, 3 for Group B and 4 for Group C, respectively; and, essentially, were the first scanning shots made by the operator. However, 20 scanning shots for each group were finally performed and evaluated.

## 4. Discussion

The restorative finishing line of full crowns can be designed according to various geometries, mainly horizontally or vertically oriented, and as shoulder, chamfer, and knife edge preparations, with mixed typologies based on the angulation of the marginal zone. When a partial crown is prepared for an esthetic restoration a horizontal margin is usually prepared, such as a shoulder design, with a sharp external angle. The presence of this sharp angle facilitates the check of the distance between the finish line and adjacent tooth, as well as the distance between the finish line and the soft tissues. However, the preparation of an abutment for a digital impression must consider limitations due to the digital impression device [23].

Based on the results of this clinical trial, the null hypothesis, that there was no difference in the capability of the IOS independent of the vertical position of the prepared finish line, was rejected (*p* < 0.005). It was pointed out that the deeper into the sulcus the position of the margin is, more of the part of the prepared root will be lost during the digital impression.

Several clinical parameters were kept under control to ensure uniformity in order to reduce the risk of bias in this RCT. All the soft tissues around preparation margins were in similarly healthy condition; the operator was a long-time experienced user of IOS and each patient received detailed instructions before performing the digital impression.

The accuracy of digital impression systems has been extensively studied in recent years [20,23]. However, the wide majority of studies were performed in vitro and designed to detect differences among different scanners [23].

The problem is that the in vitro laboratory conditions often differ from real, daily clinical situations [24]. The clinical use of IOS can be heavily complicated by factors such as: humidity of the oral environment, saliva flow, soft tissue presence and health condition, possible movements of the patient, scanning procedure and technique, limited access of the scanning probe to posterior teeth (for instance, hampered by lips and cheeks), and the varying translucency of enamel and dentine [25]. However, the results of this study showed that when all the aforementioned factors were controlled as fully as possible during impression taking, the depth of the finishing line inside the sulcus can negatively influence the final quality and accuracy of the digital impression.

A possible explanation for this finding is related to the discrete nature of intraoral scans. Unlike conventional impressions, which record a continuous surface, digital scans sample the surface at discrete intervals. A continuous surface is then generated in the software by ‘joining the dots’ according to the “stitching” algorithm. If the sample density of information is too low relative to the topology of the region (e.g., in a small patch of the impression near the gingival crevice and containing an angular crown margin too), the generated 3D surface will not replicate the true anatomy.

The results of this study clearly pointed out limitations in taking a predictable digital impression when a margin placed 1.5–2 mm into the sulcus was used and showed the need for a coronally positioned finishing line in order to catch the margins.

It was stated that low quality of impressions and insufficient preparations were the greatest obstacles for the production of high-end dental restorations [26]. In this context, IOS seems to be a logical step to prevent many possible errors.

However, it must be considered that performing a preparation is a common procedure in general dental practice, as a necessary prerequisite for the fabrication of fixed prosthetic restoration, and influences overall success substantially. During preparation, biological and technical necessities often oppose each other and therefore sometimes make it a difficult procedure for the dentist. Additionally, in daily practice the cervical margin is often located equigingivally and/or subgingivally and the positioning of the margin can be a serious obstacle to taking a perfect digital impression [27].

When the finishing line is located in the sulcus and the IOS is used, a certain amount of prepared root can’t be captured [28]. The prepared root which is not captured in the digital impression and that remains uncovered by the margin of the crown will be covered by a long epithelium attachment the same type of periodontal attachment formed after scaling and root planning [29].

The skill of the operator and the role of temporary crowns may help to address margins positioned more in depth into the sulcus.

However, few scientific data are available regarding the capability of IOS to catch margins located deeply into the sulcus. Consequently, the results of this randomized clinical trial strongly suggest the use of IOS in combination with supragingival preparations only.

It has to be emphasized that only one IOS device has been evaluated in this study; therefore, these results cannot be directly translated to other trials using different IOS devices. Similar clinical studies with a wider number of IOS are desirable.

## 5. Conclusions

Based on the results of this clinical study, the following conclusions can be drawn:

1. The deeper the position of the finishing line into the sulcus, it is more difficult to capture the margin using IOS.

2. Digital impression is not recommended when crowns’ margins are positioned deep (1.5–2 mm) into the sulcus.

## Figures and Tables

**Table 1:** Statistical results for the mean distance of the prepared root that cannot be detected with the digital impression compared to the conventional one.

*n* = 20	Juxtagengival Margins Group A	Subgengival Margins (within 1.5 mm) Group B	Deepest Margins (1.5–2.0 mm) Group C
Interproximal margins	0	0.682	0.964
Buccal margins	0	0.483	0.750

**Table 2:** The analysis performed on a site level (mesial/distal/vestibular) on the depth of both vertical preparations.

Site	Mean	Std. Dev.	Freq.
Bmesial	0.66	0.27	20
Bdistal	0.73	0.28	20
Bbuccal	0.48	0.12	20
Cmesial	1.01	0.30	20
Cdistal	0.92	0.27	20
Cbuccal	0.78	0.15	20
Total	0.76	0.29	120

One-way measure site, bonferroni tabulate: B (Group B) and C (Group C). Bmesial: Mesial site group B, Bdistal: Distal site group B; Bbuccal: Buccal site group B; Cmesial: Mesial site group C; Cdistal: dDistal site group C; Cbuccal: Buccal site group C; Std.Dev: Standard Deviation; Freq.:Frequency.

**Table 3:** Analysis of variance.

Source	SS	df	MS	F	Prob > F
Between groups	3.55666457	5	0.711332913	12.15	0.0000
Within groups	6.6717138	114	0.058523805		
Total	10.2283784	119	0.058523805		

Bartlett’s test for equal variances: chi^2^(5) = 21.8654 Prob > chi^2^ = 0.001.

**Table 4:** A post hoc analysis (Bonferroni).

Row Mean-| Column Mean	Bmesial	Bdistal	Bbuccal	Cmesial	Cdistal
Bdistal	0.07				
	1.000				
Bbuccal	−0.18	−0.24			
	0.357	0.027			
Cmesial	0.36	0.29	0.53		
	0.000	0.004	0.000		
Cdistal	0.26	0.19	0.44	−0.10	
	0.014	0.211	0.000	1.000	
Cbuccal	0.12	0.05	0.30	−0.23	−0.14
	1.000	1.000	0.002	0.045	1.000

Measure, by depth. Two-sample *t* test with equal variances.

**Table 5:** Statistical data about differences between the two types of impressions.

Group	Obs	Mean	Std. Err.	Std. Dev	[95% Confidence.	Interval]
Group B	60	0.62295	0.0325056	0.2517873	0.5579064	0.6879936
Group C	60	0.9044833	0.0340743	0.263938	0.8363009	0.9726658
Combined	120	0.7637167	0.0267633	0.293177	0.7107227	0.8167106
Difference		−0.2815333	0.0470921		−0.3747886	−0.1882781

diff = mean(Group B)−mean(Group C); *t* = −5.9784. Ho: diff = 0 degrees of freedom = 118 Ha: diff < 0 Ha: diff = 0 Ha: diff > 0. Pr(T < *t*) = 0.0000 Pr(|T| > |*t*|) = 0.0000 Pr(T > *t*) = 1.0000; Std.Err—standard error; Std.Dev—standard deviation.

## Data Availability

The data presented in this study are available on request from the corresponding author. The data are not publicly available due to privacy of patients.
